# Electrochemical Sensing of Serotonin by a Modified MnO_2_-Graphene Electrode

**DOI:** 10.3390/bios10040033

**Published:** 2020-04-02

**Authors:** Lavanya Nehru, Sekar Chinnathambi, Enza Fazio, Fortunato Neri, Salvatore Gianluca Leonardi, Anna Bonavita, Giovanni Neri

**Affiliations:** 1 Department of Bioelectronics and Biosensors, Alagappa University, Karaikudi 630003, India; 2 Department of Engineering, University of Messina, 98166 Messina, Italy; leonardis@unime.it (S.G.L.); abonavita@unime.it (A.B.); gneri@unime.it (G.N.); 3 Department of Mathematical and Computational Sciences, Physics and Earth Physics, University of Messina, 98166 Messina, Italy; enfazio@unime.it (E.F.); fneri@unime.it (F.N.)

**Keywords:** MnO_2_-graphene composite, electrode material, serotonin sensor

## Abstract

The development of MnO_2_-graphene (MnO_2_-GR) composite by microwave irradiation method and its application as an electrode material for the selective determination of serotonin (SE), popularly known as “happy chemical”, is reported. Anchoring MnO_2_ nanoparticles on graphene, yielded MnO_2_-GR composite with a large surface area, improved electron transport, high conductivity and numerous channels for rapid diffusion of electrolyte ions. The composite was characterized by X-ray photoelectron spectroscopy (XPS), Raman spectroscopy and scanning electron microscopy (SEM) for assessing the actual composition, structure and morphology. The MnO_2_-GR composite modified glassy carbon electrode (GCE) exhibited an excellent electrochemical activity towards the detection of SE in phosphate buffer saline (PBS) at physiological pH of 7.0. Under optimum conditions, the modified electrode could be applied to the quantification of serotonin by square wave voltammetry over a wide linear range of 0.1 to 800 µM with the lowest detection limit of 10 nM (S/N = 3). The newly fabricated sensor also exhibited attractive features such as good anti-interference ability, high reproducibility and long-term stability.

## 1. Introduction

5-hydroxytryptamine (5-HT), also named serotonin (SE), is one of the most important monoamine neurotransmitters that has a popular image as a contributor to feelings of wellbeing and happiness [1]. The level of serotonin in the human physiological system regulates sleeping, eating and digesting [2] and also controls the mood disorders, depression symptoms such as anxiety or depression [3,4,5]. Therefore, accurate detection of SE is of utmost importance for the diagnosis, prognosis and outcome of depressed patients. Currently, various methods like fluorometry, HPLC, coulometry, electrophoresis and ELISA have been proposed to determine the concentration of serotonin [6,7,8,9]. However, these conventional techniques are not appropriate for rapid detection and everyday tests of SE detection in real samples, because they are time-consuming, expensive and need of specialized operators. Hence, the development of simpler and cheaper electrochemical sensors for the determination of SE has received considerable interest last few years [10,11].

Many electrochemical strategies have been therefore reported for selective and simultaneous determination of SE along with several potential biological interferences [12]. For example, ascorbic acid (AA) and dopamine (DA) coexist with SE in physiological samples such as blood and urine as well as in extracellular fluid at a high concentration level. Further, a poor response and great interferences were observed at the conventional electrodes in SE determination due to their almost similar oxidation potential [13]. In order to overcome these limitations, nanostructured metal oxide [14], WO_3_ [15], Fe_3_O_4_-chitosin [16], graphene-polyaniline [17], Au-Ag nano-alloy [18] and carbon nanotube [19] have been used as electrode materials for selective and sensitive detection of SE. These modifiers are generally used to lower overpotential towards the oxidation of serotonin, in comparison with unmodified electrodes. The analytical responses obtained by using modifiers are usually higher and reproducibility of the electrode performance gets improved significantly.

MnO_2_ is one of the most promising transition metal oxides for electrochemical applications due to its non-toxicity, environmental compatibility and low cost [20,21]. However, its relatively poor electrical conductivity usually demands the addition of conductive supportive matrix (like graphene) to enhance the charge transfer rate. Graphene, as a two-dimensional carbonaceous material, is the most accredited in the field of electrochemical sensors due to its strong interaction and increased contact area which greatly promote the electrocatalyst stability and give a high electrical conductivity [22]. Recently, much research has been focused on coupling of MnO_2_ with graphene to form hybrid material with enhanced performance as compared to their individual counterparts. Thus, the integration of MnO_2_ nanoparticles on graphene sheet potentially paves the way to enhance specific surface area, improved electron transport, high conductivity and numerous channels for diffusion of electrolyte ions. Such MnO_2_-graphene composite has proved its superiority as electrode material in various applications such as solar cells, supercapacitors, and flexible electronic devices [23]. 

Here, we report an eco-friendly, rapid and economical route to synthesize MnO_2_-GR composite and its application for the fabrication of electrochemical serotonin sensor. Microwave heating increases the rate of certain chemical reactions by several folds when compared to conventional heating. Moreover, it does not produce any green gas or other side products and the use of solvents in the chemical reaction can also be removed or reduced significantly. In this context, the prepared MnO_2_-GR composite has more advantages than other nanocomposites [13,14,15,16]. This composite has been used as an electrode material for determination of SE with high sensitivity and selectivity over a wide range (0.1–800 μM) with the lowest detection limit of 10 nM. The sensor exhibits high reproducibility and efficient anti-interference ability in the presence of common interferents such as ascorbic acid and uric acid.

## 2. Materials and Methods

### 2.1. Reagents and Materials 

Analytical grade KMnO_4_, H_2_SO_4_ and NH_4_OH were purchased from Fischer Scientific, Mumbai and graphite powder (99.9995% purity), serotonin, dopamine, ascorbic acid, and uric acid were obtained from Sigma Aldrich. 0.1 M phosphate buffer solution (PBS) with different pH values were freshly prepared by adjusting pH with 0.1 M HCl or NaOH. The serotonin solution was prepared daily by dissolving the appropriate amount in PBS.

### 2.2. Synthesis of Graphene Oxide (GO) and MnO_2_-Graphene Composite

Graphene oxide was prepared from natural graphite powder according to Hummers method [24]. MnO_2_-graphene composite was synthesized by microwave irradiation method. Initially, graphene oxide (GO-50 mg) was dispersed in 100 mL of Milli-Q water and ultra sonicated for 30 min and then 0.1 M KMnO_4_ powder was added into the solution under stirring. Subsequently, the resulting suspension was kept in microwave oven at 600 W for 10 min, and then cooled to room temperature naturally. The sample was centrifuged at 3000 rpm for 30 min and dried for 12 hrs at 100 °C in ambient air after washing with ethanol and deionized water. The pure MnO_2_ nanoparticles were also prepared without adding GO in the mixture solution.

### 2.3. MnO_2_-Graphene Composite Modified Electrode

Prior to the fabrication, glassy carbon electrodes (GCEs) were polished with alumina powders (1.0, 0.5 and 0.3 µm) and then rinsed with ethanol and distilled water and dried at room temperature. An amount of 1 mg of MnO_2_-GR composite suspension was prepared (1 mg in 1 mL of water) and then 5 μL of the suspension was drop coated on the electrode, to form the MnO_2_-GR/GCE after during in ambient temperature. 

### 2.4. Instruments and Measurements

Field emission-scanning electron microscope (FE-SEM) images were obtained using a ZEISS 1540XB equipped with an EDX detector. X-ray photoemission spectra (XPS) were performed by a K-α spectrometer equipped with a conventional Al-Kα X-ray source and a concentric hemispherical analyzer with pass energy of 20 eV. During each measurement, the analysis chamber pressure was in the 10^−9^ mbar range. For all the investigated core levels, the Ag 3d_5/2_ level was used as a reference (368.0 eV). The relative concentration of manganese, oxygen and carbon atoms were calculated by the areas under the Mn 2p, O 1s and C 1s peaks weighted by the relative sensitivity factors. Raman spectra were analyzed by aXploRA micro-Raman equipment coupled with an Olympus BX40 microscope. The 532 nm line of a diode laser was focused on the samples surface through the 50X objective of the microscope. The scattered signal was analyzed by a monochromator equipped with a 600 line/mm holographic grating and collected by a CCD sensor. 

Electrochemical measurements were performed in conventional three-electrode system comprising a glassy carbon electrode (GCE; 3 mm in diameter) coated with MnO_2_-GR/GCE as the working electrode, a platinum wire as the counter electrode, and an Ag/AgCl (3 M KCl) as the reference electrode using a CHI 900 workstation. Square wave voltammetry (SWV) measurements were carried out in PBS (pH 7.0) in the potential region from 0.1 to 1.0 V with frequency of 10 Hz, an amplitude of 50 mV and a step potential of 5 mV. 

## 3. Results

### 3.1. Morphological, Raman and XPS Studies

**Figure 1:**
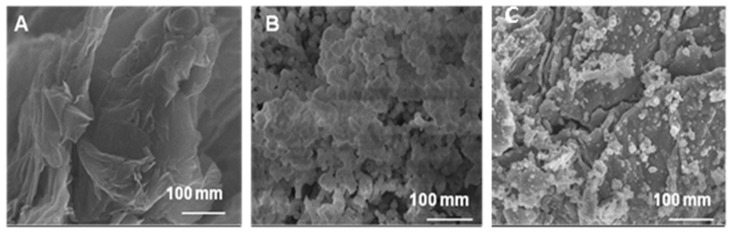
Images of (**A**) GO; (**B**) MnO_2_ and (**C**) MnO_2_-GR nanocomposite.

The surface morphology of the MnO_2_-GR composite was examined using FE-SEM. Representative images (Figure 1) show wrinkles and folds with the thin layers of graphene whereas MnO_2_ appear to self-assemble into a mesh-like structure (Figure 1B). SEM images of the composite (Figure 1C) show that the MnO_2_ particles are uniformly anchored on graphene sheets, which is a desirable prerequisite for improved electrocatalytic activity. 

**Figure 2:**
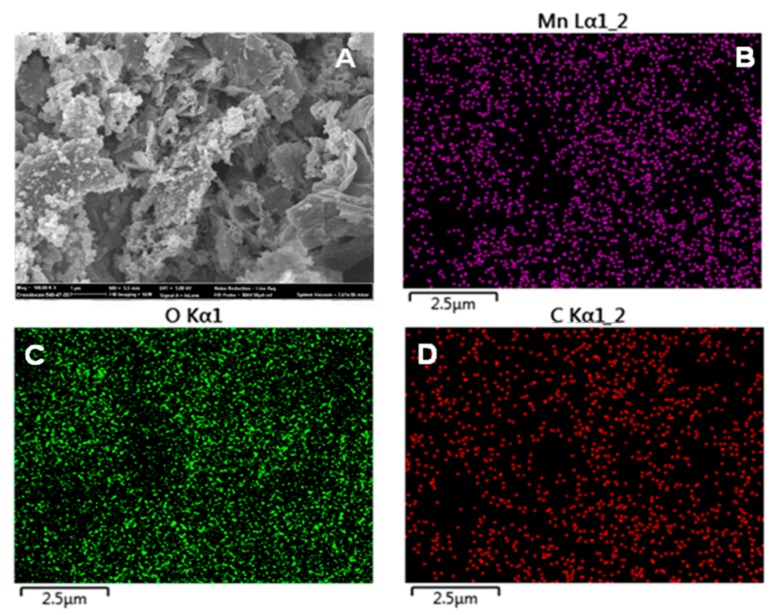
SEM-EDAX mapping of the surface of MnO_2_-GR modified electrode; (**A**) SEM image of MnO_2_-GR nanocomposite and EDAX mapping of the distribution of main elements (**B**) O; (**C**) Mn; (**D**) C.

Figure 2 shows EDAX elemental mappings with a typical SEM image beside the corresponding C, Mn and O maps. The homogeneous distribution of Mn and O elements indicates that the MnO_2_ is dispersed uniformly on graphene sheets. This wrapped MnO_2_ on graphene is expected to exhibit a higher electrical conductivity and an improved electrocatalytic activity. 

**Figure 3:**
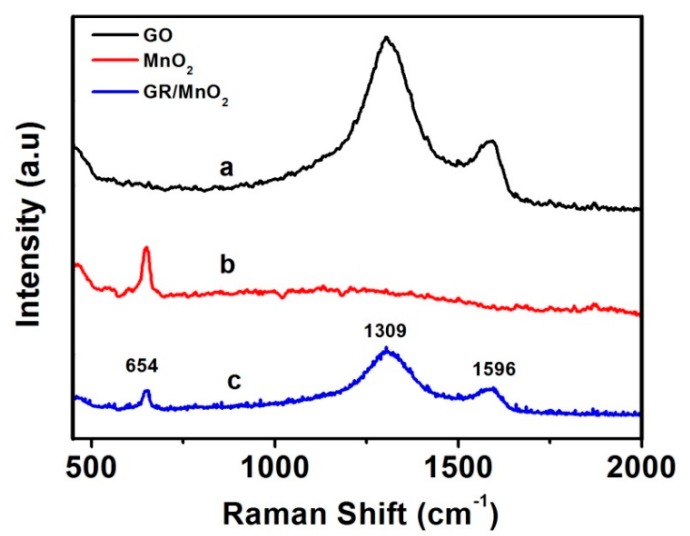
Raman spectra (a) GO, (b) MnO_2_ and (c) MnO_2_-GR nanocomposite.

Raman spectra of GO, MnO_2_ and MnO_2_-GR composite are shown in Figure 3. The characteristic MnO_2_ Raman peak centred at 654 cm^−1^, ascribed to Mn–O symmetric stretching vibration in the basal plane of MnO_6_ [25], is noted both in MnO_2_ and MnO_2_-GR composite. The peak observed for GO at 1596 cm^−1^, which is attributed to the bond stretching of the pairs of C sp^2^ atoms (known as E_2_g phonon mode) and the breathing mode of rings (A1g phonon mode) at 1309 cm^−1^, are labelled G and D bands, respectively. The I_D_/I_G_ ratio values of GO and MnO_2_-GR composite were about 1.21 and 1.09 respectively. It is noteworthy that with the addition of MnO_2_ on graphene, the I_D_/I_G_ intensity ratio decreases. As is well known, the I_D_/I_G_ intensity ratio is a measure of disorder degree, therefore its decrease suggest that the MnO_2_-GR composite is more ordered than pristine GO. This confirms the transformation of GO sheets into graphene. The reduction of GO to graphene during the preparation of MnO_2_/graphene composites has been reported also by other authors [26].

**Figure 4:**
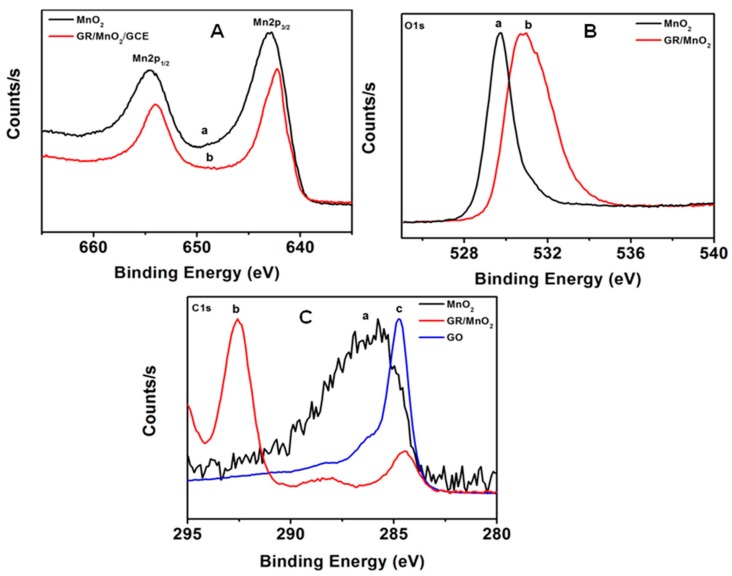
High-resolution XPS spectra of MnO_2_-GR composite (**A**) Mn 2p; (**B**) O 1s and (**C**) C 1s.

The chemical composition and surface bonding configurations were investigated by XPS analysis. The high-resolution Mn 2p XPS spectra of the pristine MnO_2_ and MnO_2_-GR nanocomposite are shown in Figure 4. Both the samples are characterized by bands typical of Mn 2p_3/2_ (642.1 eV) and Mn 2p_1/2_ (653.7 eV) respectively. Interestingly, in MnO_2_-GR, these bands are shifted to higher binding energies (BE). Instead, the spin-orbit energy separation was found similar to pristine MnO_2_ (11.6 eV), which suggests the presence of MnO_2_ nanostructures in the composite.

In the oxygen region, a broad O 1s band underlies two sub-bands. One is centred at about 530 eV related to oxygen bonded with Mn (Mn-O) in MnO_2_ crystal lattice [27] and the second, at 531 eV, can be attributed to surface hydroxyl on carbon, respectively). As regards carbon region, C 1s band come from different contributions: C-C bond at 284.5 eV, C-OH (hydroxyl and epoxy groups) bond at 285.8 eV, C-O-C and C=O bonds at 286.6 and 287.7 eV and OH-C=O (carbonyl groups) bond at 288.9 eV. The additional band at 291.0 eV refers to π–π bonds while the aliphatic carbon from the carboxylic acid functionalized graphene bonds are located in the range 292–295 eV. 

### 3.2. Electrochemical Behaviour of SE at the MnO_2_-GR Modified GCE

**Figure 5:**
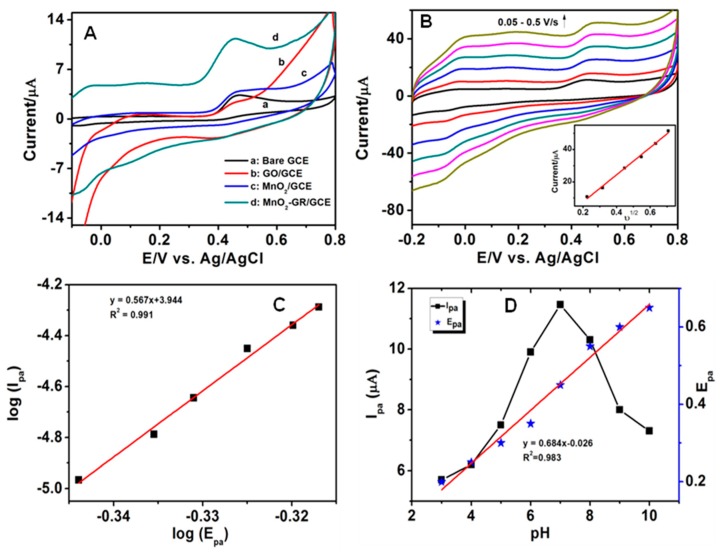
(**A**) CVs of (a) bare GCE, (**b**) GO/GCE, (**c**) MnO_2_/GCE and (**d**) MnO_2_-GR modified GCE in 100 μM SE; (**B**) CVs of MnO_2_-GR/GCE in the presence of SE at scan rates of 50–500 mV/s (**C**) log E_pa_ vs. log I_pa_ and (**D**) pH effect on the anodic peak current and peak potential of SE.

The electrocatalytic activity of serotonin at different modified electrodes was investigated by cyclic voltammetry (CV). Figure 5A shows the CVs of the bare GCE (a), GO (b), MnO_2_ (c) and MnO_2_-GR (d) modified GCEs in 0.1 M PBS (pH 7.0) containing 0.5 mM SE recorded at the scan rate of 50 mVs^−1^. As can be seen from Figure 5Aa, bare GCE shows weak anodic peak for SE indicating poor electron transfer kinetics at the interface. Compared with the bare electrode and GO/GCE, the electrochemical response of SE at the MnO_2_ modified GCE was higher with enhanced anodic peak current values of Ipa = 3.91 μA. However, a significantly higher current was noted at the oxidation potential of 0.45 V for the MnO_2_-GR composite modified GCE (curve d) corresponding to electrochemical oxidation of serotonin. The oxidation peak current (11.26 μA) of SE at MnO_2_-GR composite was two-fold higher than that of MnO_2_ and GO modified electrodes which could be mainly due to the synergistic effect of graphene and MnO_2_ NPs. 

The unique physical and chemical properties of the composite significantly improve the oxidation of SE at the modified electrode and greatly improve the current response. Here, the improved surface area of the nanocomposite provides a microenvironment for preserving the adsorbed SE molecules and therefore, MnO_2_-GR modified GCE was used for further experiments.

### 3.3. Effect of Scan Rate and Mechanisms for Electrode Reaction of SE

In order to study the plausible mechanism involved in the electrocatalytic oxidation, the effect of scan rate (υ) on the reaction of 100 µM SE at the MnO_2_-GR modified GCE have been carried out for various scan rates from 50 to 500 mVs^−1^. As shown in Figure 5B, the oxidation peak current increased linearly and the oxidation peak potential shifted slightly toward positive direction which is a typical characteristic of irreversible process. The plots of peak current as a function of square root of scan rate (υ^1/2^) for SE is shown in the inset of Figure 5B. It was noted that, the oxidation peak currents increase linearly with square root of the scan rate for various scan rates. The linear regression equation is deduced as i_pa_ (μA) = 8.413 + 3.03υ^1/2^ (mVs^−1^) (R^2^ = 0.993). Thus, the electron transfer kinetics of SE on the MnO_2_-GR/GCE was deduced. The anodic peak potential (E_p_) and log υ showed a linear relationship with the regression equations as E_pa_(V) = 0.567logυ + 3.944 (R^2^ = 0.988) for serotonin. The surface concentration of the electroactive species was estimated according to the equation,

I_p_ = n^2^F^2^AΓv/4RT

Here, the peak current is related to the surface concentration of the electroactive species Γ, where n represents the number of electrons involved in the reaction, A is the surface area of the electrode (0.07 cm^2^) and Γ is the surface coverage and the other symbols have their usual meanings. From the slope of the anodic currents vs. the scan rate, the calculated surface concentration of MnO_2_-GR/GCE is 1.037 nM cm^−2^ (n = 2) which seems to be optimum value for improved electron transfer reaction at the interface.

### 3.4. Effect of pH Value

**Scheme 1:**
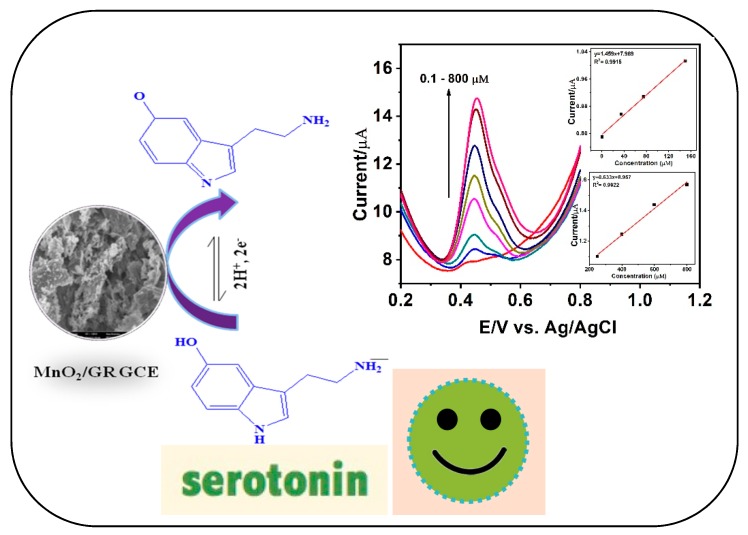
Plausible mechanism of SE detection at MnO_2_-GR/GCE.

It is well known that electrochemical reactions occurring at metal oxide electrodes are generally accompanied by the exchange of protons with the solution. The influence of pH on the electrochemical responses of MnO_2_-GR modified GCE towards the detection of SE has been investigated (Figure 5D) in different pH phosphate buffer saline (range from 4 to 9) at a scan rate of 50 mV/s. As observed in Figure 5D, the anodic peak potential (E_pa_) shifted negatively with increasing pH values, indicating that the protons participate in the electrochemical reaction of SE. A good linear relationship between E_pa_ and pH was observed with regression equation of E_pa_ (SE) = 0.068 pH + 0.026. The slope value of d_EP_/d_pH_ plot was found to be close to the theoretical value of 59 mV/pH expected for the 1H^+^ per electron stoichiometry. Therefore, the reaction of SE at the modified electrode was a two-electron process. On the other hand, the oxidation peak current (I_pa_) of SE increased with an increase of pH. The plausible mechanism for the determination of SE at the MnO_2_/GR modified electrode is shown in Scheme 1. The highest anodic peak current was observed at pH 7.0, which has been chosen for further electrochemical measurements.

### 3.5. Electrochemical Determination of SE at the MnO_2_-GR Modified Electrode

**Figure 6:**
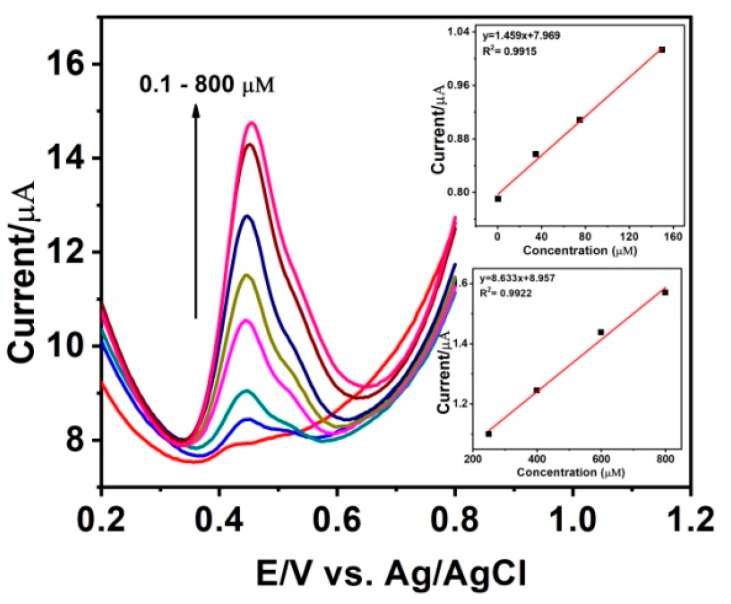
SWVs obtained for various concentrations of SE at MnO_2_-GR/GCE in 0.1 M PBS; inset shows corresponding calibration curve of SE (0.1–150 and 150–800 μM).

Square wave voltammetry (SWV) technique has been used to examine the electrochemical response of MnO_2_-GR modified GCE by changing the concentration of serotonin in the presence of ascorbic acid (Figure 6). Compared to cyclic voltammetry, the SWV technique has a lot of advantages, such as the increase of sensitivity and resolution, in quantitative analysis of physiological samples where serotonin usually coexists with higher concentrations of ascorbic acid (AA), uric acid (UA) and folic acid (FA) which strongly affects the selectivity and sensitivity [14]. In the present work, the oxidation peak current increased linearly with increasing the concentration of SE from 0.1 to 800 μM at MnO_2_-GR/GCE. The inset of Figure 6 shows the calibration curve of the modified electrode with good correlation coefficient of 0.991 and the lowest detection limit was calculated as 10 nM. Compared with the other reported sensors [15,28], the proposed electrode works at pH 7.0 without using enzymes or mediators and that the sensor has wide linear response range, low detection limit and high repeatability and stability. Anithaa et al. [15] have reported a significantly high LOD of 0.001 μM and wider linear range for the detection of SE at pH 7.0. However, the authors used gamma ray source for the electrode material modification which is a fairly sophisticated facility requiring trained manpower to operate. Similarly, Lavanya et al. also reported SnO_2_-SnS_2_ composite electrode material for SE detection with high accuracy [28]. The disadvantage here is the relatively less stable SnS_2_ when compared to SnO_2_. Hence, there was a need to develop a less expensive, more stable electrode material with superior sensing ability for detection of the very important happy chemical serotonin. In this endeavor, we have noted that the present electrode made of MnO_2_ along with the highly stable graphene exhibited wider concentration of 0.1–150 and 150–800 μM when compared to the other electrodes (Table 1). SWV experiments for the electrochemical determination of SE at the MnO_2_ and GO modified GCEs individually did not yield the desired results in comparison with that of MnO_2_-GR composite (results not shown). Hence, it can be concluded that the MnO_2_-GR modified electrode is more suitable for the determination of SE in both biological samples and pharmaceutical products.

### 3.6. Selectivity, Stability and Reproducibility of the MnO_2_-GR/GCE

**Figure 7:**
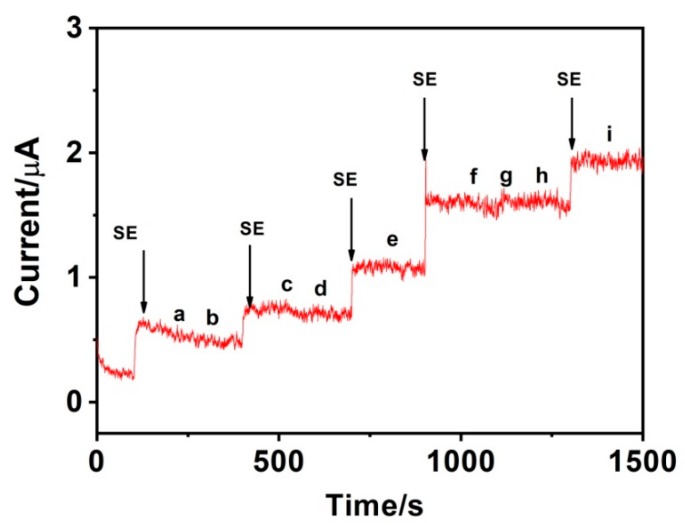
Amperometric response of the MnO_2_-GR modified GCE for the addition of 50 µM SE and successive addition of interferents 500 μM each in the sequence of (a) dopamine, (b) epinephrine, (c) folic acid, (d) uric acid, (e) ascorbic acid, (f) glucose (g) Na^2+^, (h) K^+^, and (i) Mg^2+^ in 0.1 M PBS.

Selective determination of serotonin is a challenge because its oxidation potential is at the same value as that of other neurotransmitters such as dopamine (DA) and epinephrine (EP). Anithaa et al. found that the oxidation potential of SE (0.39 V) is close to that of DA (0.22 V) and EP (0.20 V) on WO_3_ nanoparticles [15]. Similar findings have been reported by other authors, with the result of the overlapping of oxidation potentials towards detection of SE in mixed samples [16]. Hence, influence of some potential inorganic ions and organic molecules on the electrochemical determination of SE has been tested in order to evaluate the anti-interference ability of the fabricated sensor (Figure 7). The results indicated that 100 fold concentrations of Na^2+^, K^+^, Mg^2+^ and 10-fold excess dopamine, epinephrine, folic acid, uric acid, ascorbic acid, glucose (500 μM) had no obvious influences on the response of 50 µM serotonin with deviations below ± 5%. Moreover, the oxidation potential of NE (0.6 V) is far from SE (0.4 V), therefore the interferent NE will not influence the detection SE.

Moreover, the stability and reproducibility of the MnO_2_-GR/GCE were also evaluated in 100 μM SE in PBS. The oxidation peak current of the developed electrode had a relative standard deviation (RSD) of 1.29% after 20 successive scans, indicating the MnO_2_-GR/GCE has good stability. In addition, it was noted that the modified electrode easily gets regenerated for the next measurement even after using it for a continuous three cycles in 0.1 M PBS, and a RSD value of about 2.3% obtained for the regenerated MnO_2_-GR/GCE after measurement for 10 times in the same solution. The reproducibility of the developed sensor was studied with six different electrodes and the peak current obtained in the five repeated measurements showed an RSD value of 1.2%. The results clearly indicate that the developed sensor has high selectivity, reproducibility and good stability.

### 3.7. Real sample Analysis

**Figure 8:**
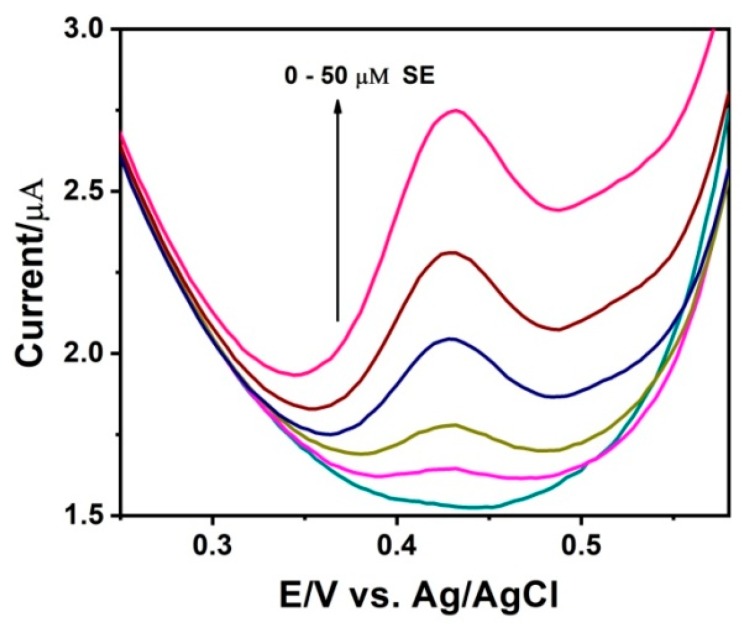
SWVs obtained for blood serum (green line) and after successive addition of SE concentrations to blood serum at the MnO_2_-GR/GCE in 0.1 M PBS.

To evaluate the applicability of the MnO_2_-GR/GCE, the human blood serum was used for real sample analysis. Abnormal concentrations of SE in serum have been shown to reflect the derailed serotonergic function in central nervous system. As a preliminary application in clinical studies, the developed sensor was used to assess the SE concentration in healthy human serum samples diluted 100 times with a 0.1 M PBS (pH 7.0) before the measurements. Figure 8 shows voltammograms of unspiked and spiked samples. The oxidation peak current at 0.42 V was increased when adding the known concentration of SE. The recovery results are given in Table 2.

## 4. Conclusions

In this work, MnO_2_-GR nanocomposite has been synthesized by microwave irradiation method and used as electrode material for the fabrication of novel electrochemical sensor for the determination of serotonin in the presence of ascorbic acid for the first time. The developed electrode showed improved electrocatalytic activity towards the detection of SE when compared to other modified electrodes in phosphate buffer saline at pH 7.0. Moreover, the fabricated sensor exhibited wide linear range from 0.1 to 800 μM for SE with the lowest detection limit of 10 nM with good selectivity, high reproducibility and stability. The analytical application of the developed sensor was examined with human blood serum for quantification of SE and which yielded acceptable results. These results demonstrate that the MnO_2_-GR nanocomposite has great potential for applications in electrochemical sensors and biosensors design.

## Figures and Tables

**Table 1:** Comparison of previous electrodes for the determination of SE with MnO_2_-GR/GCE.

Electrode	Method	Linear Range (μM)	LOD (μM)	Ref.
^a^ WO_3_/GCE	DPV	0.01–600	0.001	[15]
^b^ Fe_2_O_3_-CHI/GCE	DPV	0.1–100	0.08	[16]
^c^ GR-PANI/GCE	DPV	0.2–10	0.011	[17]
^d^ Au-Ag/GCE	DPV	0.002–4.8	0.0016	[18]
^e^ SnO_2_-SnS_2_/GCE	SWV	0.1–700	0.045	[28]
^g^ Acetylcholine/GCE	DPV	0.8–200	0.8	[29]
^f^ GR-NS/GCE	DPV	1-100	0.038	[30]
^h^ ** MnO_2_-GR/GCE**	**SWV**	**0.1–800**	**0.01**	**This work**

^a^ γ-WO_3_ nanoparticles modified glassy carbon electrode; ^b^ Fe_2_O_3_-Chitosan modified glassy carbon electrode; ^c^ Graphene polyaniline modified glassy carbon electrode; ^d^ Ag-Au modified glassy carbon electrode; ^e^ SnO_2_-SnS_2_ modified glassy carbon electrode; ^f^ Graphene nanosheet modified glassy carbon electrode; ^g^ Acetylcholine modified glassy carbon electrode, ^h^ MnO_2_-Graphene modified glassy carbon electrode.

**Table 2:** Determination of SE in human blood serum sample using MnO_2_-GR/GCE.

Sample	SE Concentration (μM)		Recovery (n = 3) (%)
	Added	Found	
1	1	0.9	101.3
2	5	5.2	97.9
3	10	10.3	98.3
4	15	14.6	102.6
5	20	20.3	101.8
